# Bacterial steroid hydroxylases: enzyme classes, their functions and comparison of their catalytic mechanisms

**DOI:** 10.1007/s00253-018-9239-3

**Published:** 2018-07-21

**Authors:** Maciej Szaleniec, Agnieszka M. Wojtkiewicz, Rita Bernhardt, Tomasz Borowski, Marina Donova

**Affiliations:** 1 Jerzy Haber Institute of Catalysis and Surface Chemistry, Polish Academy of Sciences, Niezapominajek 8, 30-239 Kraków, Poland; 2 Lehrstuhl für Biochemie, Universität des Saarlandes, Campus B2 2, 66123 Saarbrücken, Germany; 3 G.K. Skryabin Institute of Biochemistry and Physiology of Microorganisms, Russian Academy of Sciences, Pushchino, Moscow Oblast 142290 Russia

**Keywords:** Steroid hydroxylation, Cytochrome P450, 3-ketosteroid 9α-hydroxylase, Steroid C25 dehydrogenase

## Abstract

The steroid superfamily includes a wide range of compounds that are essential for living organisms of the animal and plant kingdoms. Structural modifications of steroids highly affect their biological activity. In this review, we focus on hydroxylation of steroids by bacterial hydroxylases, which take part in steroid catabolic pathways and play an important role in steroid degradation. We compare three distinct classes of metalloenzymes responsible for aerobic or anaerobic hydroxylation of steroids, namely: cytochrome P450, Rieske-type monooxygenase 3-ketosteroid 9α-hydroxylase, and molybdenum-containing steroid C25 dehydrogenases. We analyze the available literature data on reactivity, regioselectivity, and potential application of these enzymes in organic synthesis of hydroxysteroids. Moreover, we describe mechanistic hypotheses proposed for all three classes of enzymes along with experimental and theoretical evidences, which have provided grounds for their formulation. In case of the 3-ketosteroid 9α-hydroxylase, such a mechanistic hypothesis is formulated for the first time in the literature based on studies conducted for other Rieske monooxygenases. Finally, we provide comparative analysis of similarities and differences in the reaction mechanisms utilized by bacterial steroid hydroxylases.

## Introduction

### Role of hydroxylations in aerobic and anaerobic steroid metabolism

Steroids (Greek, *ster*eos = s*o*l*ids*) represent a specific class of terpenoid lipids that contain a gonane core of four fused cycloalkane rings (A–D). The steroid superfamily includes various structures such as sterols (e.g., cholesterol, sitosterol, ergosterol); bile acids; corticoids; cardiac aglycones; vitamin D; and insect molting hormones. Multiple functions of steroids are essential for living organisms of the animal and plant kingdoms (Baker 2011).

Structural modifications of steroids highly affect their biological activity. Hydroxylation results in increase of polarity of the hydrophobic steroid molecules, affects their toxicity, translocation through the cell envelope, and greatly influences their biological effects. Positions of the hydroxyl groups and stereochemistry around carbons to which they are attached in the cycloalkane rings, as well as in the side chain of steroids, are of importance. For example, the presence of hydroxyl function in position 11β is essential for anti-inflammatory activity (e.g., cortisol, prednisolone) (Fegan et al. 2008), the 16α-hydroxyl function is of importance for synthetic glucocorticoids such as triamcinolone and dexamethasone (Berrie et al. 1999), the 14β-hydroxyl group is typically found in cardioactive steroids (Ali Shah et al. 2013), the 7-hydroxylated derivatives of dehydroepiandrosterone (DHEA) and epiandrosterone (EpiA) have neuroprotective effects (Milecka-Tronina et al. 2014; Wojtal et al. 2006), and the 1α- and 25α-hydroxyl functions are of significance for the vitamin D_3_ (VD3) activity (Bikle 2014; Prosser and Jones 2004). 25-hydroxycholesterol was shown to exhibit antiviral activity towards a broad spectrum of viruses by activating interferons, immune cells, and increasing the production of immune mediators (Blanc et al. 2013; Gold et al. 2014; Liu et al. 2013).

Hydroxylation of steroids by diverse bacteria is considered mainly as a prelude to their catabolism as carbon and energy sources. Recent data confirmed that steroid-degrading bacteria are globally distributed and prevalent in wastewater treatment plants, soil, plant rhizospheres, and the marine environment (Holert et al. 2018). Two hundred sixty-five putative steroid degraders have been identified only within *Actinobacteria* and *Proteobacteria*, whose genomes are available in NCBI (NCBI Rf_Seq) (Bergstrand et al. 2016). Except for *Actinobacteria* and *Proteobacteria*, a more recent metagenomics investigation suggested that steroid degraders may be present in other bacterial groups; several alpha- and gammaproteobacterial lineages not previously known to degrade steroids (Holert et al. 2018).

**Fig. 1:**
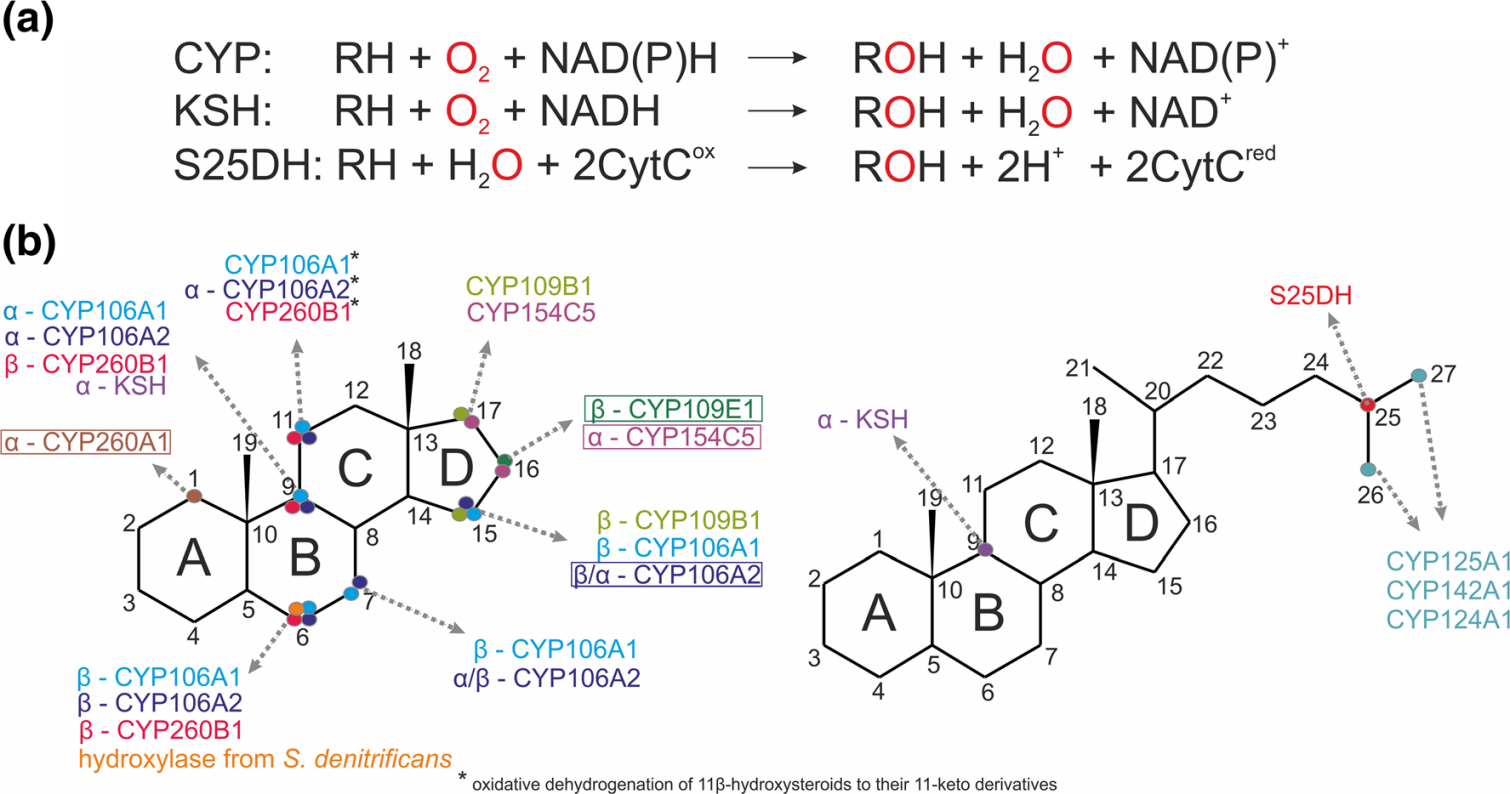
Hydroxylation of steroids by bacterial enzymes. **a** Reaction schemes for CYP, KSH, and S25DH. **b** Regioselectivity of hydroxylations reported for aerobic cytochrome P450 family represented by: CYP106A1 *Bacillus megaterium* DSM319, CYP106A2 *B. megaterium* ATCC13368 (Kiss et al. 2015c), CYP109B1 *B. subtilis* 168 (Agematu et al. 2006), CYP109E1 *B. megaterium* DSM19 (Jozwik et al. 2016), CYP154C5 *N. farcinica* IFM 10152 (Bracco et al. 2013), CYP260A1, CYP260B1 from *Sorangium cellulosum* So ce56 (Litzenburger and Bernhardt 2016) CYP125, CYP142, CYP124 from *M. tuberculosis* CDC1551 or H37Rv (Johnston et al. 2010), non-heme KSH from *Actinobacteria* (Petrusma et al. 2014), and anaerobic S25DH from *Sterolibacterium denitricans* (Dermer and Fuchs 2012)

Bacterial hydroxylases, being a part of steroid catabolic pathways, play an important role in steroid degradation. They participate in both cholesterol and cholate degradation pathways by initiating the degradation of the aliphatic side chain or the opening of the sterane ring. Depending on the oxic or anoxic environment, different catalytic strategies are realized (Fig. 1). The aerobic bacterial degradation of the side chain of cholesterol and other sterols generally occurs through the mechanism which is similar to the β-oxidation of fatty acids and proceeds via CoA thioester intermediates (see e.g., Galán et al. 2017 for a review). At least three steps of this reaction cascade are catalyzed by cytochromes P450s, such as CYP125, CYP142, and CYP124 and they are an initial reaction, oxyfunctionalization at the ω-position at C26 (27) to form the terminal alcohol and sequential oxidations to aldehyde and carboxylic acid (Frank et al. 2015; Johnston et al. 2010; Ouellet et al. 2011). Orthologs of these enzymes have been identified in all sterol-transforming *Actinobacteria*, whose genomes are available in the databases, e.g., *Mycobacterium tuberculosis* (Mtb) H37Rv; *Mycobacterium neoaurum* NRRL 3805B; *M. neoaurum* VKM Ac-1815D, 1817D; *Mycobacterium smegmatis* mc2 155; *Rhodococcus jostii* RHA1; *Gordonia neofelifaecis* NRRL B-59395; and *Nocardioides simplex* VKM Ac-2033D (Capyk et al. 2009b; Garcia-Fernandez et al. 2013; McLean et al. 2009; Ouellet et al. 2011; Rosloniec et al. 2009; Shtratnikova et al. 2016). It is of importance that these P450 enzymes selectively and regiospecifically oxidize the relatively unreactive terminal methyl group, but not the more reactive tertiary carbon (C25) of the sidechain (Ouellet et al. 2011).

Aerobic hydroxylation of the C25-tertiary carbon atom was reported for *Pseudonocardia autotrophica*, *Streptomyces griseolus*, and *Sebekia benihana*. The reaction results in 25-hydroxyvitamin D_3_ and 1α,25-dihydroxyvitamin D_3_ (Ban et al. 2014; Sawada et al. 2004). The VD3-specific hydroxylases, Vdh in *P. autotrophica*, CYP105A1 in *S. griseolus*, and CYP-sb3a, a member of the bacterial CYP107 family in *S. benihana*, were shown to perform double hydroxylations at both positions 25 and 1α. These reactions are of great importance for the production of physiologically active forms of VD3. Hydroxylation at the C25-tertiary atom of the diosgenin F-ring to form the spirosteroid isonuatigenone has been reported for the soil-dwelling actinomycete strain *Streptomyces virginiae* IBL-14 (Wang et al. 2007; Wang et al. 2009).

Under anaerobic conditions, the hydroxylation of steroids seems to be less frequent. Ten years ago, a new class of molybdopterin containing steroid hydroxylases has been discovered in a denitrifying β-proteobacterium *Sterolibacterium denitrificans* Chol-1S that is capable of anaerobic mineralization of cholesterol (Chiang et al. 2008b). After initial oxidation of cholesterol to cholest-4-en-3-one by the dehydrogenase/isomerase anaerobic cholesterol metabolism enzyme A (AcmA), the steroid is either hydroxylated at C25 of the side chain by the molybdenum-containing steroid C25 dehydrogenase (S25DH) (Dermer and Fuchs 2012) or first oxidized to cholest-1,4-dien-3-one by a flavin-cholest-4-en-3-one Δ^1^-dehydrogenase (AcmB) (Chiang et al. 2008a), and then hydroxylated at C25 (Chiang et al. 2007; Chiang et al. 2008b; Warnke et al. 2017) (Fig. 1). Based on genomics and metabolomics studies, *S. denitrificans* appeared to utilize a range of steroid C25 dehydrogenases for oxygen-independent C25-hydroxylations of steroids with different isoprenoid side chains, followed by an unusual conversion to C26-oic acids (Warnke et al. 2017). This hypothesis was very recently confirmed by successful heterologous overexpression of paralogous S25DH-like genes in *Thauera aromatica* K172 and characterization of substrate specificity for four different enzymes (Jacoby et al. 2018). After complete degradation of the aliphatic side chain, androst-4-en-3,17-dione (AD) and/or androst-1,4-dien-3,17-dione (ADD) enter the central degradation pathway via 2,3-*seco*-AB-rings opening (Chiang and Wael 2011; Wang et al. 2013; Wang et al. 2014). One more hydroxylation in anaerobic cholesterol degradation pathway was observed in the presence of 3-mercaptopropionate. However, the enzyme/s involved in this 6-hydroxylation of testosterone are still unknown (Wang et al. 2014). To date, only for S25DH, the biotechnological applications have been established for the hydroxylation of the cholesterol derivate and, most importantly, the production of 25-hydroxy vitamin D_3_ (calcifediol or calcidiol) (Rugor et al. 2017a).

Yet another type of steroid hydroxylases, Rieske monooxygenases (e.g., 3-ketosteroid 9α-hydroxylase, KSH), are involved in the so-called 9,10-seco pathway which is so far the only known aerobic route for bacterial degradation of the steroid core. The KSH enzymes were best characterized for *Rhodococcus* and *Mycobacterium* species and they represent two-component monooxygenases, composed of an oxygenase (KshA) and a flavin-dependent ferredoxin reductase (KshB) unit (Bragin et al. 2013; Capyk et al. 2009b; Guevara et al. 2017; Hu et al. 2010; Penfield et al. 2014; Petrusma et al. 2011; Petrusma et al. 2012; Petrusma et al. 2014). Along with 3-ketosteroid-1(2)-dehydrogenases (KstD), KSH is responsible for the cleavage of the 9(10)-C-C bond to form 9(10)-secosteroids with an aromatized A-ring. Deletion mutations of KstD or KshA/KshB resulted in the production of valuable androstane steroids such as 9α-hydroxyandrostenedione (9-OH-AD), or ADD, respectively, as major products from phytosterol (Galan et al. 2017; Garcia-Fernandez et al. 2017; Yao et al. 2014). Deletion of both, KstD and KSH, enables production of AD (Galan et al. 2017). These approaches are widely exploited for the generation of industrial whole cell biocatalysts for the pharmaceutical industry, since AD, ADD and 9-OH-AD are the key starting molecules for multiple chemical syntheses of therapeutic steroids.

The 9,10-secosteroids are further hydroxylated by flavin monooxygenases. The 3-hydroxy-9,10-secoandrosta-1,3,5(10)-triene-9,17-dione (3-HSA), which is formed spontaneously after 9α-hydroxylation of ADD, is hydroxylated at C4 to 3,4-dihydroxy-9,10-secoandrosta-1,3,5(10)-triene-9,17-dione (3,4-DHSA) by TesA1A2 complex in the testosterone degradation pathway of *Comamonas testosteroni* (Horinouchi et al. 2004; Horinouchi et al. 2012) or by HsaAB in the cholesterol degradation pathway in *M. tuberculosis* (Dresen et al. 2010). These enzymes are composed of two components: (i) flavin-dependent monooxygenase responsible for binding of the reduced flavin, O_2_, and a secosteroid substrate which results with activation of oxygen and hydroxylation of the substrate (HsaA and probably TesA1) and (ii) flavin-reducing component (HsaB) responsible for binding of the oxidized flavin and its reduction with NADH (Chen et al. 2017; Dresen et al. 2010). The topic of flavin-dependent monooxygenases has been covered in other reviews (Huijbers et al. 2014; Sucharitakul et al. 2014).

Apart from hydroxylases involved in the steroid catabolic pathways, various bacterial hydroxylases, which are known to oxidize other organic molecules such as terpenes, were shown to catalyze steroid conversions (Janocha et al. 2015) (see below). The broad functionality spectrum of bacterial P450s from *Streptomyces coelicolor* A3(2), *Bacillus subtilis* 168, *Bradyrhizobium japonicum* USDA110, *Nostoc* sp. PCC 7120, and *Nocardia farcinica* IFM 10152 was demonstrated by Agematu et al. (Agematu et al. 2006).

Bacteria of different taxonomic positions have been reported to carry out allylic hydroxylation to epimeric 7-alcohols such as 7α- and 7β-hydroxy dehydroepiandrosterone (DHEA) (Donova 2007; Mahato and Garai 1997). 7α-hydroxylase activity towards lithocholic (LCA) and deoxycholic (DCA) acids was described for *Actinobacteria* of *Amycolatopsis*, *Lentzea*, *Pseudonocardia*, and *Saccharopolyspora* genera. New strains capable of 7α-hydroxylation of DCA were revealed also among *Nocardiopsis*, *Nonomuraea*, and *Saccharothrix* species (Deshcherevskaya et al. 2016; Kollerov et al. 2013). The reactions are of importance for biotechnological production of valuable cholic acids (e.g., ursodeoxycholic, ursocholic acids). Recently, the formation of 1α-hydroxycorticosterone has been described for intracellular *rhodococci* within the renal-inter-renal tissue of the winter skate *Leucoraja ocellata* (Wiens et al. 2017). This kind of activity has not been described before towards corticosteroids. The presence of hydroxylase activity towards different steroids was reported for different bacterial species (Donova 2007), but in some cases, the corresponding genes and the enzymes are still to be identified and characterized. Targeted mutagenesis of bacterial P450s enables novel hydroxylation reactions of steroids to form commercially valuable compounds such as 16α-hydroxynandrolone and 1α-hydroxyandrostenedione (e.g., Liu and Kong 2017; Venkataraman et al. 2015).

The examples given above illustrate the prospects of bacterial hydroxylases for structural modification of steroids (Fig. 1). Their catalytic versatility, activity, and solubility provide a platform for multiple applications in biotechnology. Being overexpressed, they will stimulate higher biotransformation rates, thus allowing to overcome major bottlenecks for industrial applications such as low biocatalyst activities, insufficient selectivities, and space-time yields. The knowledge of the role and features of steroid hydroxylases from steroid catabolic pathways contributes to the metabolic engineering of novel effective biocatalysts as well as it helps to identify new targets for drug development.

In this review, we highlight the biochemistry, mechanism of action, functionality, and structures of the basic known types of bacterial hydroxylases: cytochrome P450s, Rieske monooxygenases, and molybdopterin dehydrogenases (Fig. 1). For the first time, the hypothesis describing reaction mechanisms proposed for all above-mentioned steroid hydroxylases are compared and general mechanistic conclusions are drawn. Finally, the role of the enzymes and perspectives of their application in biotechnology and medicine are discussed.

## Cytochrome P450-dependent steroid hydroxylation

Aerobic hydroxylations of steroids can be performed by cytochromes P450 belonging to the class of monooxygenases. While such reactions with steroids as substrates were among the first ones discovered for this class of enzymes (Cooper et al. 1963), the discovery of bacterial P450s being able to catalyze steroid hydroxylation started only in the middle of the 70s (Berg et al. 1975). Cytochromes P450 are hemoproteins catalyzing, in general, the hydroxylation of aromatic and aliphatic substrates (Bernhardt and Urlacher 2014). In addition, N-, S-, O-demethylation; N- or S-oxidation; C–C bond cleavage; and many other reaction types have been described and the substrates also include, besides steroids, alkanes, fatty acids, terpenes and antibiotics.

### Cytochrome P450 reaction mechanism

**Fig. 2:**
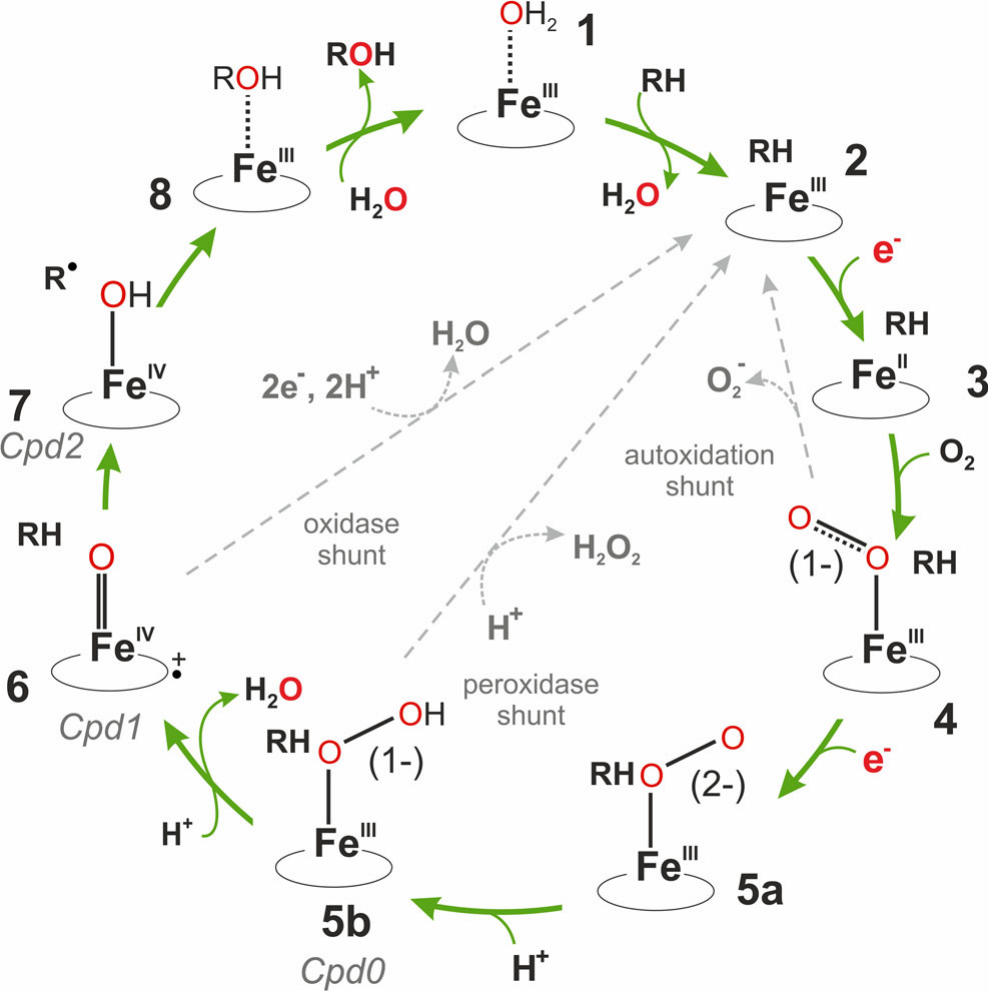
Reaction scheme of cytochrome P450. The numbers (1–7) represent the actual state of the enzyme: 1 = low-spin substrate-free state. 2 = high-spin enzyme-substrate complex. 3 = high-spin ferrous state. 4 = oxy-ferrous state. 5a = ferric peroxo intermediate. 5b = ferric hydroperoxo intermediate/compound 0. 6 = High-valent iron-oxo state/compound I. 7 = Fe(IV)hydroxide (compound II)/radical intermediate. 8 = product coordinately bound to the ferric resting state. RH and ROH illustrate the substrate and the product, respectively. The three unproductive shunt-pathways are marked with dashed gray arrows and the reduced oxygen products are shown as outlets. (The cycle has been adapted from Denisov et al. (Denisov et al. 2005)

For the hydroxylation, one atom of the molecular oxygen is incorporated into the organic product, whereas the second one gets reduced to water. Activation of the molecular oxygen takes place on the heme iron using electrons provided by NAD(P)H and delivered via an electron transfer chain. The basic reaction mechanism has been elucidated and is shown in Fig. 2.

In brief, the first step shows that binding of the substrate to the resting form of the enzyme, which has a water-coordinated low-spin ferric heme iron, causes displacement of the water ligand, and thereby a change to the five-coordinate high-spin enzyme-substrate complex (2). In the next step the first electron is delivered by a corresponding redox partner and it facilitates binding of molecular oxygen to the high-spin ferrous iron (3). Thus, formed ferric superoxo state (4) is relatively stable and can be reduced by a second electron to form a nucleophilic ferric peroxo state (5a). This state is normally short-lived and readily converted to the ferric hydroperoxo intermediate (5b) by addition of a proton. This intermediate is also called compound 0. Addition of a second proton to the terminal oxygen takes place during heterolytic cleavage of the O–O bond, which leads to the formation of a highly reactive ferryl heme π-cation radical (6), the so-called compound I, and a water molecule. Finally, the oxygen atom is incorporated into the substrate by a “hydrogen abstraction/oxygen rebound” mechanism (6 → 7 → 8) forming the hydroxylated product and the ferric resting state (8). A highly basic Fe(IV)hydroxide (compound II) serves as an intermediate in this reaction (Green et al. 2004; Mak and Denisov 2018). As also shown in Fig. 2, electrons can be consumed unproductively in three uncoupling reactions leading to the formation of a superoxide anion radical, hydrogen peroxide, or water.

The electron transport chain in bacterial P450 systems often contains a FAD-containing NAD(P)H-dependent reductase and a ferredoxin of the 2Fe-2S type. However, bacterial P450s were shown to use a multitude of other redox systems (for review, see Hannemann et al. 2007). Therefore, it can be tedious to identify a working redox system for a novel bacterial P450. However, some redox systems are ubiquitous and universal (e.g., mammalian NADPH-dependent adrenodoxin); hence, they can serve as first choice for a successful reconstitution of an enzymatic activity (Ewen et al. 2012).

### Bacterial cytochromes P450 involved in cholesterol degradation

#### CYP125 family

The best-studied member of this family, CYP125A1, comes from *M. tuberculosis*. It is involved in the degradation of cholesterol, which, in turn, plays an important role in the growth of this bacterium and in phagocytosis (for reviews, see Ortiz de Montellano 2018). As found by knockout studies in *R. jostii* RHA1 (Rosloniec et al. 2009), as well as in vitro investigations using a purified enzyme, CYP125A1 catalyzes the 26-hydroxylation of cholesterol and cholest-4-en-3-one (Capyk et al. 2009a; McLean et al. 2009). The 3D structure of the enzyme has been solved (Johnston et al. 2010) and it provides insight into the structure of this important enzyme. In *M. smegmatis*, CYP125A3 and CYP125A4 were identified, which are also able to oxidize cholesterol (Ortiz de Montellano 2018).

#### CYP142 family

In addition to CYP125A1, also CYP142A1 of *M. tuberculosis* was shown to be a cholesterol 26-hydroxylase (Driscoll et al. 2010). Heterologously expressed CYP142A2 from *M. smegmatis* also catalyzes the oxidation of cholesterol and cholest-4-en-3-one to their 26-carboxylic acid derivatives (Garcia-Fernandez et al. 2013). The crystal structure of this protein as a complex with cholest-4-en-3-one gave insight into the structural differences between this protein and CYP125A1 that manifest in different configurations of the products (R- or S-configuration) (Johnston et al. 2010).

### Bacterial cytochromes P450 involved in hydroxylation of steroids

So far, only a limited number of bacterial steroid hydroxylases have been described. Starting in the ‘70s with more detailed investigations of CYP106A2 from *Bacillus megaterium* ATCC13368, later on, other bacterial P450s came into focus and were analyzed concerning their ability to convert steroids. Thus, 213 bacterial P450s were expressed in *Escherichia coli* and investigated concerning their ability to hydroxylate testosterone (Agematu et al. 2006). It was found that only 24 of them were able to produce products, mainly hydroxylated at the β-face. Using a screening system based on FT-ICR/MS, 29 P450 ORFs from seven *Bacillus* strains have been studied (Furuya et al. 2009). Five P450s from *Bacillus cereus* were analyzed in more detail and steroid conversion was shown for families CYP106, CYP107, and CYP109. In a preceding report, the authors also demonstrated steroid conversion by CYP134A1 from *B. subtilis* (Furuya et al. 2008). However, the products have not been identified in these reports.

More recently, besides *Bacillus* P450s, also cytochromes P450 from other bacteria, such as *Sorangium cellulosum* and *Nocardia*, have been analyzed concerning their ability to bind and convert steroids (Bracco et al. 2013; Khatri et al. 2016; Salamanca-Pinzon et al. 2016).

#### CYP106A family

CYP106A2 from *B. megaterium* ATCC13368 was the first bacterial steroid hydroxylase being purified and characterized (Berg et al. 1975). As summarized in a very recent review (Schmitz et al. 2018), it hydroxylates a multitude of steroidal compounds—such as progesterone, testosterone, cortisol, and 11-deoxycorticosterone—mainly at the 15β-position. Side reactions have been observed for some of the substrates at the 11α-, 6β-, and 9α- positions (Kang et al. 2004; Lisurek et al. 2004). Interestingly, when using 3-hydroxy instead of 3-keto steroids, as in the case of pregnenolone and DHEA, hydroxylation takes place mainly at the 7β-position. Prednisone and dexamethasone are also hydroxylated in 15β-position giving rise to possible new routes for drug synthesis (Putkaradze et al. 2017). Moreover, besides substrates with gonane structure (as steroids), those also displaying the ursane, abietane and dammarane type can be converted by CYP106A2 (summarized by (Schmitz et al. 2018). Recently, another member of the same family and subfamily, CYP106A1 from *B. megaterium* DSM 319, displaying 63% amino acid sequence identity with CYP106A2, has been characterized, demonstrating a very similar substrate and product profile as CYP106A2 (Kiss et al. 2015a; Kiss et al. 2015c).

The crystal structure of CYP106A2 from *B. megaterium* as well as from a marine bacterium, *Bacillus* sp. PAMC 23377, has been solved recently and provides a valuable tool for the rational redesign of this enzyme (Janocha et al. 2016; Kim et al. 2017). CYP106A2-dependent steroid conversion was successfully investigated and applied in *Pseudomonas putida* S12, *E. coli*, and *B. megaterium* based whole-cell systems (Hannemann et al. 2006; Ruijssenaars et al. 2007; Schmitz et al. 2014; Zehentgruber et al. 2010). After optimization, yields of 3.7 g/L/day for progesterone, 5.5 g/L/day for testosterone, and 2.7 g/L/day for DHEA conversion were obtained as theoretical space-time productivity calculated from the initial reaction rates. The stability and activity of CYP106A2 with cyproterone acetate was also tested under process conditions using the enzyme overexpressed in *B. megaterium* MS941 and demonstrates the applicability of this system for biotechnological purpose (Kiss et al. 2015b).

Although CYP106A2 already demonstrates a broad range of substrate conversion, the activity and selectivity of this enzyme are highly tunable using site-directed mutagenesis and molecular evolution (for review, see Schmitz et al. 2018). Based on computer modeling or the 3D structure of CYP106A2 and site-directed mutagenesis and/or molecular evolution approaches, the selectivity of hydroxylation was successfully shifted from the 15β- to the 11α-, 9α-, or 6β-positions of progesterone (Lisurek et al. 2008; Nguyen et al. 2012; Nikolaus et al. 2017).

#### CYP109 family

The CYP109 family of genes is prevalent among different bacterial species (htpp://drnelson.utmem.edu/CytochromeP450.html). CYP109B1, which was shown to convert testosterone mainly to the 15β-OH product, with some small reactivity towards C17 (Agematu et al. 2006), is one of the well-studied bacterial CYPs, which might be due to its broad substrate specificity (Girhard et al. 2010). Its 3D structure was solved quite recently in a substrate-free open conformation (Zhang et al. 2015). Another member of the CYP109 family, CYP109E1 from *B. megaterium* DSM319, has been characterized very recently as a steroid binding and converting enzyme and its structure has been solved in substrate-free and substrate-bound forms (Jozwik et al. 2016). The enzyme revealed a highly dynamic active site being wide open in the substrate-free and corticosterone-bound (binds but is not converted) form and closed when bound to its substrate testosterone, which is hydroxylated at the 16β-position. MD simulations demonstrated that testosterone can be bound in an unproductive conformation as shown in the crystal as well as in an about 180° reversed conformation bringing C16 close to the heme iron.

#### CYP154 family

First indications for steroid hydroxylations by P450s of this family were obtained by Agematu et al. using genome profiling (Agematu et al. 2006). The authors reported that CYP154C5 from *N. farcinica* IFM 10152 highly selectively converted testosterone into 16α-OH testosterone. This reaction is of special interest for the pharmaceutical industry and thus further potential substrates have been tested and successful biotransformations were observed for pregnanes and androstanes, such as pregnenolone, DHEA, progesterone, AD, and nandrolone (Bracco et al. 2013). The hydroxylation took place exclusively at the 16α-position and a whole-cell system was established allowing product formation on a preparative scale with a total turnover number (TTN) (μmol substrate consumed μmol^−1^ CYP154C5) exceeding 2000. The crystal structures together with four different substrates (pregnenolone, progesterone, testosterone, AD) have been solved (Herzog et al. 2014). It was shown that the steroid substrates bind to the active site in such a way that only C16 is in close enough proximity to the heme to allow hydroxylation. A comprehensive comparison of the active sites of CYP1091 and CYP154C5 hydroxylating testosterone at the 16β- and the 16α-position, respectively, is given in (Jozwik et al. 2016). It was pointed out that the shape and volume of the active sites were significantly different in both proteins. The different shapes of the active sites lead to a roughly perpendicular orientation of the steroid relative to the heme in CYP109E1, which is optimal for the C–H abstraction from the β-face, with a parallel binding in the case of CYP154C5 bringing the α-face of C16 close to the heme.

Moreover, 27 putative P450s from *Streptococcus griseus* have been expressed and analyzed concerning their activities. It was found that cells producing CYP154C3, an isoform of CYP154C5, were also able to hydroxylate various steroids (testosterone, progesterone, DHEA, and others) at the 16α-position (Makino et al. 2014).

#### CYP260 family

Very recently, two members of the CYP260 family from *S. cellulosum* Soce56 have been identified as potent and highly selective steroid hydroxylases (Khatri et al. 2016; Salamanca-Pinzon et al. 2016). It was demonstrated that CYP260A1 is able to convert various C19 steroids like testosterone, AD, and their derivatives at the 1α-position, identifying this way the first bacterial P450 catalyzing hydroxylation at this position. In contrast, CYP260B1 was identified as a 6β-hydroxylase of 11-deoxycortisol (cortodoxon). Moreover, also other Δ4 C21 steroids (progesterone, 17OH-progesterone, 11-deoxycorticosterone, cortisol, cortisone) were converted (with different selectivities) as well as dexamathasone and betamethasone. Interestingly, when using 11-deoxycorticosterone as substrate, hydroxylation at position 9α was found (Litzenburger and Bernhardt 2017) giving rise to the suggestion that the absence of the OH group at position 17α allows the substrate to move into the active site of CYP260B1 in another orientation than 11-deoxycortisol. This is in accordance with the results of docking studies that used the crystal structure of CYP260B1 as a docking target. The crystal structures of both proteins were solved in substrate-free forms (Khatri et al. 2016; Salamanca-Pinzon et al. 2016). In addition, conversion of a Δ4 C21 steroid, 11-deoxycorticosterone, by CYP260A1 was demonstrated and was further improved with respect to activity and selectivity by replacing Ser326 with asparagine (Khatri et al. 2016).

It was demonstrated very recently that the concentration ratio between CYP260A1 and its redox partners does not only affect the catalytic activity but also the product pattern (Khatri et al. 2017). The reason for this behavior is not yet clear, but it might explain differences in selectivities observed in in vitro and in vivo studies (Nguyen et al. 2012). Further investigations, also using electrochemically driven catalysis (Kuzikov et al. 2016), might be able to shed light into this process. Here, comparisons of structural parameters of the protein-protein complexes with the electron transfer pathways and properties could give hints about the interplay between electron transfer rate, substrate mobility within the active site, and hydroxylation velocity and selectivity.

A big step to a deeper understanding of the structural basis for the regioselectivity of steroid hydroxylation by bacterial P450s comes from a very recent study where the 3D structures of CYP260A1 mutants have been solved together with the substrate. A mutant with the serine residue S276 replaced by asparagine converted progesterone predominantly into 1α-OH progesterone, while the S276I mutant lead to the 17α-OH product. The structures of the mutants revealed two alternate binding modes of progesterone in the active site of CYP260A1 and fully explained the observed product specificities (Khatri et al. 2018).

Taken together, it seems that the active sites of bacterial P450s are quite variable, allowing positioning of steroids in different ways to be optimal for hydroxylation at various C-atoms as well as the α- or β-face. The steroid can be bound either in a parallel or perpendicular orientation relative to the heme and it can also be bound in different ways concerning the location of the A- and D-ring. The data obtained so far give valuable information for further rational redesign aiming at either changing and/or improving the stereo- and regioselectivity of steroid hydroxylation by different bacterial P450s.

## Non-heme O_2_-dependent hydroxylation

### Rieske monooxygenase

3-ketosteroid 9α-hydroxylase (KSH) is a key enzyme of the 9-10-seco central degradation pathway of steroids and it has been known since late 60s. (Gibson et al. 1966). The enzyme was discovered both in pathogenic and environmental *Actinobacteria* such as *Mycobacterium* (Brzostek et al. 2005; van der Geize et al. 2007; Wovcha et al. 1978), *Nocardia* (Strijewski 1982), *Rhodococcus* (Datcheva et al. 1989; Geize et al. 2002; Petrusma et al. 2009), *Arthrobacter* (Dutta et al. 1992), as well as in proteobacteria such as *C. testosteroni (*Horinouchi et al. 2012*).* Recently, a wide comparative genomic analysis revealed the presence of KSH genes in new genera such as *Acinoplanes*, *Shewanella*, and *Pseudoalteromonas* (Bergstrand et al. 2016). KSH is a multimeric two-component class IA monooxygenase consisting of a terminal oxygenase (KshA) and a ferredoxin reductase (KshB). It requires reduced pyridine nucleotides as an electron donor for molecular oxygen activation and exhibits higher activity with NADH than NADPH (Fig. 1a).

### Structure and reactivity

**Fig. 3:**
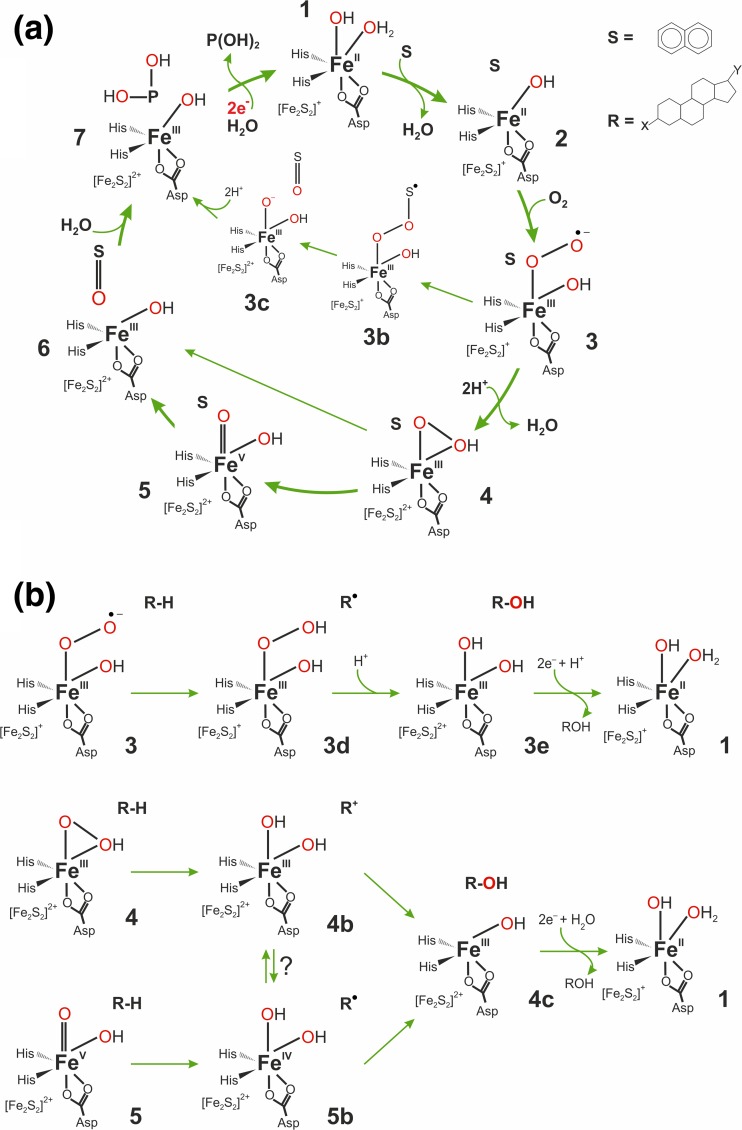
The mechanism proposed for Rieske-type oxygenases. **a** The mechanism for dioxygenation of aromatic compounds. **b** Compilation of various possible mechanistic pathways proposed for monooxygenation of aliphatic substrates (such as steroids hydroxylated by KSH)

To date, several structures of KshA component were solved: two from *M. tuberculosis* (PDB 2ZYL and 4QCK) (Capyk et al. 2009a) and three from *Rhodococcus rhodochrous* (PDB 4QDC, 4QDD, and 4QDF) (Penfield et al. 2014) (Capyk et al. 2009a). KshA contains N-terminal “Rieske” domain and a larger “catalytic” domain. The former harbors a Rieske-type 2Fe-2S cluster and the later a mononuclear ferrous iron coordinated by a bidentate Asp and two His residues (Fig. 3) (Capyk et al. 2009a; Penfield et al. 2014). The functional active site of KshA is formed by a head-to-tail interaction of KshA subunits within the functional homotrimer. Such arrangement places the Rieske-type 2Fe-2S and Fe centers from two different subunits in a close proximity (app. 12 Å). The electron transfer between these sites is facilitated by the conserved Asp residue, which forms H-bond interactions with His ligands of both metallocenters (Capyk et al. 2009a). The substrate preference is controlled by a 16-residue “mouth loop” located at the entrance of the active site (Petrusma et al. 2012; Petrusma et al. 2014). KshB, the reductase component, seems to be a monomeric protein (Capyk et al. 2009a) with a NAD-binding region, a plant-type 2Fe-2S cluster, and a flavin prosthetic group. Unfortunately, to date, no crystal structure of the KshB is available. It was demonstrated that the whole enzyme can be reconstituted after overexpression and both components, KshA and KshB, are required for KSH activity (Capyk et al. 2009a).

KSH catalyzes regioselective hydroxylation at the C9 atom of the ring B of ketosteroids leading to formation of 9α-hydroxy-products in the aerobic degradation pathway of cholesterol, short chain steroids, cholate, and/or bile acid (Bergstrand et al. 2016; Geize et al. 2002). The 9α-hydroxy-ADD, a product of KSH and KSTD activity, is relatively unstable. As a result a cleavage of the C–C bond between C-9 and C-10 occurs, with a concomitant aromatization of the ring A leading to formation of 3-hydroxy-9,10-*seco*-androst-1,3,5(10)-triene-9,17-dione (3-HSA) (Geize et al. 2002; Gibson et al. 1966). Initially, AD and ADD were proposed as KSH native substrates based on the analysis of metabolites in mutants with KSH gene deletion (Andor et al. 2006; Geize et al. 2002; van der Geize et al. 2008). However, multiple isoforms of the oxygenase subunit KshA were found in *Mycobacteriaceae* and *Rhodococci*, which hydroxylate different steroid derivatives with varied efficiency (Petrusma et al. 2014). For example, KshA1 from *R. rhodochrous* exhibits preference for substrates with a carboxylate side chain at C17 and seems to be involved in cholate catabolism, whereas KshA5 hydroxylates a broader range of steroid substrates: cholesterol, cholate, progesterone and AD with a kinetic preference for substrates with no C17 side chain (e.g., 4-estrendione, 5-androstandione and testosterone (Penfield et al. 2014)). KshA from *M. tuberculosis* was reported to present twofold higher substrate specificity for ADD then for AD (Capyk et al. 2009a). In the case of *R. rhodochrous*, it was demonstrated that all five KshA oxygenases can pair with the same KshB reductase, forming enzymes with distinct substrate spectrums and application ranges (Petrusma et al. 2011).

### Rieske-type oxygenases reaction mechanism

Rieske-type oxygenases were first discovered as enzymes responsible for cis-dihydroxylation of unactivated aromatic compounds and the majority of information concerning the mechanism of the catalytic cycle has been obtained for this sub-group, which includes, among others, naphthalene dioxygenase and benzoate 1,2-dioxygenase. The initial stages of the catalytic cycle are relatively well established thanks to multiple structural and spectroscopic data. Single turnover experiments by Lipscomb et al. for the oxygenase component (NDO) of naphthalene dioxygenase showed that the two electrons available within the reduced Rieske/non-heme iron pair (species 1 in Fig. 3a) are sufficient for the progress of the oxidative reaction. This is true for both the native substrate-naphthalene (Wolfe et al. 2001) as well as the mechanistic probe substrate-norcarane (Chakrabarty et al. 2007). This means that the chemical transformations (dihydroxylation of naphthalene and monohydroxylation of norcarane) can be done with only these two electrons available for dioxygen activation and that the electrons are delivered to the oxygenase component at the end of the cycle when the product is released (7 → 1 in Fig. 3). Binding of the substrate triggers a dissociation of one water ligand from the non-heme iron (Ohta et al. 2008), which may be viewed as a preparatory step for the reaction with O_2_. Besides the presence of the substrate in the active site, O_2_ binding requires that the Rieske cluster is in a reduced state (Wolfe et al. 2001; Wolfe et al. 2002). Structural studies on 2-oxoquinoline 8-monooxygenase and carbazole 1,9a-dioxygenase revealed a sequence of conformational changes that accompany this reduction and prepare the active site both for O_2_ binding and efficient electron transfer from the Rieske cluster to non-heme iron (Ashikawa et al. 2012; Martins et al. 2005). Actual binding of O_2_ to species 2 could either lead to Fe(III)-superoxo species (3 in Fig. 3), which still features the reduced Rieske cluster, as proposed for benzoate 1,2-dioxygenase (Rivard et al. 2015), or to Fe(II)-superoxo/oxidized Rieske cluster species (not shown), as suggested based on a DFT computational study (Bassan et al. 2004b). Details of further chemical steps that lie between O_2_ binding (3) and dissociation of the enzyme-product complex (**7**) are still unsettled and are the subject of ongoing studies. Available X-ray structures of tertiary enzyme-substrate-O_2_ complexes usually show O_2_ species bound to iron ion in a side-on fashion and with an O–O bond length typical for a peroxide (Ashikawa et al. 2012; Karlsson et al. 2003). These structural data suggested that a (hydro)peroxo species (4) could be a catalytically relevant intermediate and this conclusion got support from the results of single turnover experiments in which H_2_O_2_ was shown to be an efficient terminal oxidant (“peroxide shunt”) (Wolfe and Lipscomb 2003). Moreover, spectroscopic (EPR, Mössbauer) data obtained for a trapped reactive intermediate indicate it is indeed a high-spin Fe(III)-hydroperoxo species (Neibergall et al. 2007). Results of two computational DFT studies led to conflicting suggestions as to whether a high-valent Fe(V = O species (5) can be formed. In the first study, employing the B3LYP functional, a high barrier was found for the 4 → 5 step, and instead, an alternative and energetically viable pathway was proposed, whereby the aromatic ring 2-electron oxidation is coupled to heterolytic cleavage of the O–O bond, i.e., 4 → 6 (Bassan et al. 2004a). In the second study, an acceptable barrier height was obtained for the 4 → 5 step for the B97D functional and hence it was proposed that O–O bond cleavage precedes aromatic ring oxidation, i.e. the reaction follows the 4 → 5 → 6 sequence (Pabis et al. 2014). Parallel studies on biomimetic complexes showed mononuclear non-heme Fe(V)=O complexes can indeed form from Fe(III)-peroxo precursors and they are the reactive species responsible for oxidative transformations performed by these bioinspired complexes (Fan et al. 2018; Oloo and Que 2015). Yet, another reaction pathway was proposed for benzoate 1,2-dioxygenase based on results of single turnover kinetic studies (Rivard et al. 2015). Namely, the Fe(III)-bound superoxide anion is proposed to attack the aromatic ring and form a peroxo bridge between a substrate radical and the Fe(III) ion; 3 → 3b. Subsequent O–O cleavage would be facilitated by an electron transfer from the Rieske cluster; 3b → 3c.

From the above summarized plausible reaction mechanisms of Rieske oxygenases, which were formulated for cis-dihydroxylation of aromatics, one can extrapolate and propose three alternative reaction mechanisms for monohydroxylation of aliphatic substrates where the reactive species is either Fe(III)–O_2_^−•^ (3), or Fe(III)–OOH (4) or Fe(V)=O (5) (Fig. 3b). In the first scenario, superoxide anion would first abstract a hydrogen atom from the substrate forming a Fe(III)–OOH/R^•^ intermediate (3d) and then the O–O bond would be cleaved with the help of the electron provided by the Rieske cluster. Several detailed mechanism can be envisioned for this 3d → 3e step, yet the net result would be an OH radical transfer to the aliphatic radical. In the second case, a hydride would be abstracted from aliphatic carbon concertedly with a heterolytic cleavage of the O–H bond (4 → 4b). OH rebound to the aliphatic carbocation (4b → 4c) would complete the hydroxylation reaction. The third mechanism assumes the actual oxidant responsible for the C–H bond cleavage (shown here as hemolytic; 5 → 5b) is a high-valent Fe(V)=O species; rebound of the OH radical would yield the final hydroxylation product (4c). Currently, there is very little experimental evidence to discriminate between these three alternative mechanisms of aliphatic hydroxylation; only the product distribution obtained for norcarane and bicyclohexane mechanistic probes suggest that this process proceeds through radical and not carbocation intermediates (Chakrabarty et al. 2007). Definitively, more experimental and computational studies are needed to nail down the actual mechanism(s) operational for Rieske oxygenases.

### Application

The KSH system has two types of biotechnological applications. The first approach is application of KSH for regiospecific hydroxylation of steroids at the 9α-position. The feasibility of 9OH-AD production was demonstrated by co-over-expression of KSH from *M. neoaurum* JC-12 and GDH from *B. subtilis* 168 in *B. subtilis* 168 host, yielding an efficient bioconversion system (with final yield of 7.23 g/L and a conversion of 90.4%) (Zhang et al. 2016). On the other hand, knockout of the KshB gene in sterol degrading bacteria leads to production of ADD (Galan et al. 2017) as further degradation (i.e., ring B opening) is blocked. This approach was previously achieved by traditional mutation techniques. Finally, blocking of KSH by small-molecule inhibitors has a high potential as a treatment for *M. tuberculosis* infection. Such inhibitors could block the catabolic pathway and inhibit the pathogen growth (Capyk et al. 2011; Petrusma et al. 2014). Furthermore, such drugs might not only block cholesterol catabolism but could also stimulate intracellular production of reactive oxygen species and depletion of energy reserves through the futile depletion of NADH by uncoupling the catalytic cycle of KshA (Capyk et al. 2009a).

## O_2_-independent hydroxylation

### Molybdenum hydroxylases

Steroid C25 dehydrogenase (S25DH), also known as steroid C25 hydroxylase, catalyzes hydroxylation at the tertiary C25 carbon atom of cholest-4-en-3-one and/or cholest-1,4-dien-3-one. Such hydroxylation initiates the aliphatic side chain degradation of sterols, which finally yields AD and/or ADD, respectively (Lin et al. 2015; Warnke et al. 2017). The enzyme was discovered in *S. denitrificans*, a gram-negative facultative anaerobic β-proteobacterium, which has been established as a cholesterol-mineralizing model organism under anaerobic conditions (Tarlera 2003). Up to date, S25DH has been isolated only from *S. denitrificans*, although there are other known microorganisms capable of anaerobic cholesterol mineralization, e.g., partially described β-proteobacterium strain 72Chol (Harder and Probian 1997). Genome analysis of γ-proteobacterium *Steroidobacter denitrificans* revealed the presence of a S25DH-like enzyme, although *St. denitrificans* is not capable of growth on steroids featuring an aliphatic side chain (Yang et al. 2016).

S25DH is a molybdenum enzyme that belongs to the so-called EBDH-like hydroxylases (Dermer and Fuchs 2012; Heider et al. 2016), where EBDH stands for ethylbenzene dehydrogenase, the first enzyme of the class, (Heider et al. 2016; Kniemeyer and Heider 2001). Similarly to EBDH, S25DH enables a highly regioselective hydroxylation of unactivated hydrocarbons, i.e., various steroids, exclusively at the C25 atom utilizing an oxygen atom from water instead of molecular oxygen (Chiang et al. 2007; Rugor et al. 2017a; Szaleniec et al. 2014).

Up to now there is no crystal structure of S25DH. Biochemical studies revealed that it is a αβγ-heterotrimeric enzyme with molybdo-*bis*(pyranopterin guanine dinucleotide) cofactor (Mo-*bis*-MGD or Moco) in the catalytic α subunit (Heider et al. 2016; Hille 2013). Besides Moco, EBDH-like hydroxylases contain five [Fe–S] clusters (one in the α subunit, four in the β subunit) and a heme *b* in the γ subunit. All cofactors form an electron transfer conduit between Moco and the external electron acceptor, presumably a high redox potential cytochrome *c* (Kloer et al. 2006; Szaleniec et al. 2007).

In the genome of *S. denitrificans* there are seven more genes coding for putative S25DH-like α subunits, five more genes coding for S25DH-like β and γ subunits as well as two copies of specialized chaperone dedicated to Moco insertion (Heider et al. 2016; Niedzialkowska et al. 2017). Based on sequence similarity to S25dA, the putative S25DH-like isoenzymes can be divided into three groups: (i) high sequence identity (i.e., S25dA2, 82%; S25dA3, 74%; and S25dA4, 72%), (ii) low sequence identity (S25dA5, S25dA6, S25dA7—app. 38%), and (iii) sequence identity higher to the gene coding for the α subunit of p-cymene dehydrogenase (42%) (Strijkstra et al. 2014) then to S25dA (40%). Six α isoforms (S25dA2-A7) are expressed in response to the presence of particular steroids, whereas expression of S25dA8 is not increased in the presence of cholesterol or phytosterols (Dermer and Fuchs 2012; Warnke et al. 2017). Proteomic and metabolomics analyses confirmed S25dA as the main type of α subunit expressed during growth on cholesterol and S25dA3 during growth on ergosterol (Warnke et al. 2017). Therefore, it was suggested that isoenzymes of the S25DH α subunit may be used to assemble αβγ S25DH-like enzymes that catalyze similar hydroxylations, but for different sterol substrates (Heider et al. 2016). This hypothesis was very recently confirmed by successful production of recombinant S25DH isoenzymes (S25DH_1–4_) in *T. aromatica* K172 and *Azoarcus* sp. CIB and characterization of substrate specificity for individual enzymes (see below and Jacoby et al. 2018). The attempts to obtain overexpress S25DH enzymes in *E. coli* systems yielded either non or trace activity only (Jacoby et al. 2018; Rugor et al. 2017b).

### Structure and reactivity

The core of the active site is a molybdenum atom coordinated by four S^−^ ligands of the two pyranopterin guanine dinucleotides, a monodentate Asp residue, and a catalytically active oxo ligand. The six ligands of the Mo(VI) ion attain distorted octahedral geometry which, upon gradual reduction of the Mo ion, to Mo(V) and Mo(IV), changes towards distorted trigonal prismatic coordination with a decrease of the S_1_–S_2_–S_3_–S_4_ dihedral angle. Although the presence of the Mo=O moiety was not confirmed directly for S25DH, it was experimentally verified by EXAFS studies for EBDH (Graham, Szaleniec, unpublished data). The monodentate binding mode of Asp211 is ensured by a strong H-bond with Lys450, which ensures that after reduction the Mo site will retain an open coordination site to bind a water molecule. In contrast to the EBDH active site, the one in S25DH does not host a His residue that could take part in an OH rebound reaction, as was proposed for EBDH (Szaleniec et al. 2010; Warnke et al. 2017).

The homology modeling and MD simulations revealed that the S25DH binding site is most probably of cylindrical shape (25 Å long, 12 Å in diameter) with its wall lined with multiple Trp and Tyr residues. The substrate is bound predominantly by vdW interactions (app. 70%), whereas the rest of binding can be attributed to electrostatic and H-bond interactions between the C3-keto/C3-hydroxy group of the steroid and polar residues present at the entrance to the binding cavity. These electrostatic interactions seem to be crucial for a proper substrate positioning and may explain the significant difference in the relative specific activity or conversion rate between C3-keto and C3-hydroxysubtrates (Rugor et al. 2017b).

Until very recently, kinetic data for S25DH were available only for the mixture of isoenzymes obtained from the *S. denitrificans* enabling distinguishing of three substrate groups with a decreasing rate of hydroxylation: (i) 3-ketosterols, (ii) vitamin D_3_, and (iii) 3-hydroxysterols and their esters (Dermer and Fuchs 2012; Rugor et al. 2017b). Warnke et al. reported a more than threefold higher *V*_max_ for VD3 (1.48 nmol/min/mg) compared to that obtained for the 3-hydroxy steroid 7-dehydrocholesterol (0.47 nmol/min/mg) (Warnke et al. 2016). Recently, the apparent *K*_*M*_ values were determined in an electrochemical system yielding *K*_*M*_ = 740 μM for cholest-4-en-3-one and *K*_*M*_ = 327 μM for VD3 (Kalimuthu et al. 2018). The advent of overexpression system for S25DH in *T. aromatic* K172 allowed kinetic characterization of individual enzymes. It was possible to obtain the apparent kinetic constants (i.e., measured in the presence of 9% 2-hydroxypropyl-β-cyclodextrin) for S25DH_1_/cholest-4-en-3-one and VD3 (*K*_*M*_ 390 μM and 290 μM, and *k*_cat_ 1.11 and 0.23/s, respectively); S25DH_2_/cholest-4-en-3-one and 7-dehydrocholesterol (*K*_*M*_ 124 μM and 123 μM, *k*_cat_ 0.29 and 0.33/s, respectively); S25DH_3_/campest-4-en-3-one (*K*_*M*_ 1.84 mM, *k*_cat_ 0.018/s); and S25DH_4_/cholest-4-en-3-on, campest-4-en-3-one and β-sitost-4-en-3-one (*K*_*M*_ 450, 340 and 120 μM, *k*_cat_ 0.21, 0.17, and 0.14/s). These results clearly showed that *S. denitrificans* utilizes different S25DH-like enzymes for conversion of different substrates with S25DH_2_ hydroxylating 7-dehydrocholesterol and S25DH_4_ hydroxylating campest-4-en-3-one and β-sitost-4-en-3-one. The role of other S25DH-like enzyme is not yet established (Jacoby et al. 2018).

### Steroid C25 dehydrogenase reaction mechanism

**Fig. 4:**
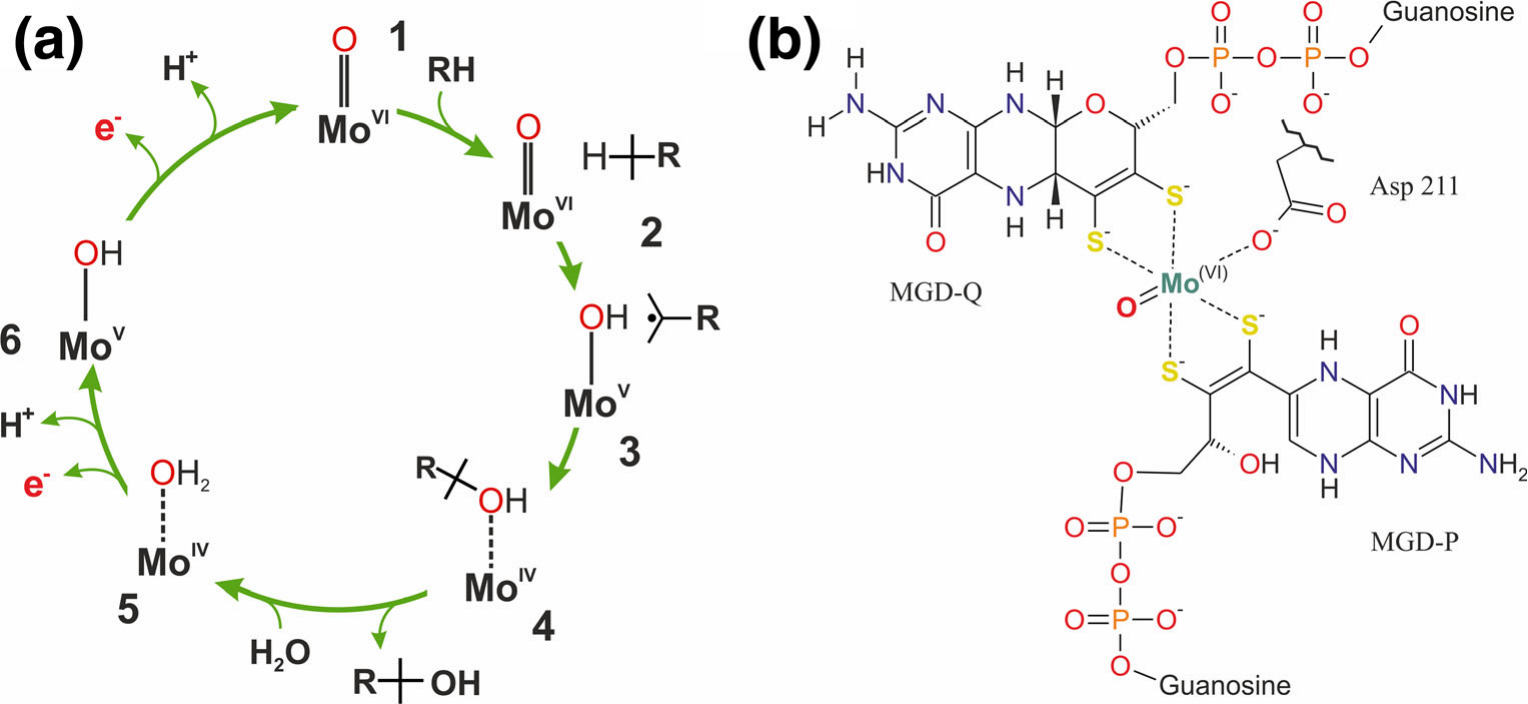
**a** The schematic representation of the mechanism proposed for S25DH. The whole cycle is composed of two parts: substrate oxidation where substrate is hydroxylated and Moco is reduced (reductive half-cycle) and enzyme re-oxidation (oxidative half-cycle) where water is bound to Moco and two electrons are transferred in two one-electron processes through the chain of FeS clusters to heme and finally to the external acceptor (putatively cyt *c*). The electron transfers are associated with proton release from the (Mo-)OH_2_ ligand to water molecules present in the active site cavity. **b** Molybdenum cofactor found in EBDH-like hydroxylases. The MGD-P ligand is shown in with the open pyran-ring as found in the EBDH structure (2IVF)

The mechanistic hypothesis for S25DH was formulated as a result of combined molecular modeling and kinetic isotope effect studies (Rugor et al. 2017b). The general steps of the reaction are similar to that proposed for EBDH (Szaleniec et al. 2014) (Fig. 4): 1 → 2 the substrate binds to the active site that contains oxidized catalytically active Moco (Mo(VI)); 2 → 3 the C–H bond is activated by Mo(VI)=O in a homolytic cleavage (TS1) and an H atom is transferred to Moco forming Mo(V)–OH and a radical intermediate product (3); 3 → 4 the OH group is rebound to the radical intermediate with concomitant reduction of molybdenum to Mo(IV) (TS2) and the alcohol product is formed, the latter is coordinated to Mo(IV) via a free electron pair of the OH group (4). In the next step (4 → 5), the product departs from the Moco and leaves the cavity and the free coordination site is used to bind a water molecule forming Mo(IV)–OH_2_ (5). This is followed by a two-step deprotonation of the Mo–OH_2_ (5 → 6 → 7) concomitant with two electron transfers from Moco to the 4Fe–4S cluster present in the α subunit and further on through the chain of FeS clusters in the β subunit to the heme *b* cofactor in the γ subunit. These electrons are finally transferred to the external electron acceptors in two 1-electron redox processes (Rugor et al. 2017b).

In contrast to the previously reported mechanism for EBDH, the intermediate product seems to feature a steroid radical species as formation of the carbocation in a heterolytic C–H cleavage is associated with higher energy barrier. As a result, unlike in the mechanism obtained for EBDH (Szaleniec et al. 2014), the TS2 barrier may be energetically close to TS1, although the observed (4.5) and calculated (6.2–7.9) KIE suggests that C–H cleavage is predominantly controlling the kinetics of the process. Other mechanistic variants, such as oxidative dehydrogenation followed by Mo–OH_2_ rebound or a bulk water attack on the dehydrogenated substituent, which would also yield an alcoholic product, were experimentally and theoretically excluded (Rugor et al. 2017b).

### Application

S25DH from *S. denitrificans* Chol-1S is expressed only in a slow growing anaerobic bacterium and an efficient overexpression system has not been available during most of the applied studies. Nevertheless, due to high regioselectivity of the catalyzed reaction and relatively easy coupling to the inexpensive reoxidants (such as potassium ferricyanide) it was proposed as a catalyst in several biocatalytic syntheses. The most interesting process developed with S25DH is hydroxylation of vitamin D_3_ (cholecalciferol) to 25-hydroxyvitamin D_3_ (calcifediol) that is currently used as a drug in several calcium or vitamin D_3_ disorders (Jean et al. 2008; Jetter et al. 2014), rickets as well as to treat the patients that are unable to activate VD3 due to decreased hepatic function (Brandi and Minisola 2013). Synthesis of 25-OH-steroids can be conducted with a crude enzyme preparation in the presence of a β-cyclodextrin solubilizer together with an organic co-solvent. This approach allows hydroxylation of VD3 with a final concentration of 1.4 g/L (3.5 mM) of calcifediol within 3 days. The potential application of S25DH for biocatalytic synthesis of calcifediol seems to be especially attractive since recent establishment of the overexpression system as recombinant S25DH1 preparation is 6.5-fold more active than wild-type extract and enables conversion of 0.38 g/L of VD3 in 3 h (Jacoby et al. 2018). The proposed system was also applied for hydroxylation of various 3-ketosteroids such as: cholest-4-en-3-one, cholest-1,4-dien-3-one and cholest-4,6-dien-3-one with a final concentration in the range of 2.2 g/L, or sterols such as: cholesterol, 7-dehydrocholesterol, with a final concentration in the range of 0.8 g/L (Rugor et al. 2017a). Purified S25DH was also efficiently immobilized on silica (Rugor et al. 2017a) or on a microfiltration membrane (Kalimuthu et al. 2018). In both cases, the enzymatic reaction was coupled to an electrochemical system, which turned out to be sensitive to changes in substrate concentration. Due to that fact S25DH can be considered as a potential element for construction of an electrochemical biosensor detecting cholecalciferol or 3-ketosteroids as an electrochemical signal (current produced in the electrode reaction coupled to the enzymatic process) differs for different concentrations of steroids in the medium (Kalimuthu et al. 2018).

## Mechanistic similarities and differences

There are several important mechanistic similarities in reactions catalyzed by CYP, Rieske-type Ksh, and molybdenum hydroxylases, despite structural differences both in their active sites and protein structures. Naturally, some of these similarities stem from the very nature of the catalyzed process, i.e., 2-electron oxidation of organic compound. As for every oxidoreductase, the whole catalytic cycle can be divided, from the perspective of the cofactor, into two phases: (i) reductive half-cycle where the substrate gets oxidized and the enzyme’s cofactor is reduced and (ii) oxidative half-cycle where the oxidative agent is reduced and the enzyme’s cofactor is oxidized. The highest similarities we can find in the reductive half-cycle as in each case the reactive metal-oxygen species has to be formed for efficient C–H activation. In case of CYP, it is the oxoferryl [(Fe^IV^=O)heme]^+•^ radical carbocation (compound I) and in case of S25DH it is the Mo^VI^=O moiety of the Moco cofactor. In case of KSH, the form of the reactive metal-oxygen species is still debated, i.e., Fe(III)–O_2_^−•^, Fe(III)–OOH, or Fe(V)=O, although in light of the results obtained for the inorganic biomimetic models it seems that Fe(V)=O is the most plausible form (Fan et al. 2018; Oloo and Que 2015). These activated oxygen ligands are able to abstract an H atom from the hydrocarbon resulting in the formation of the transient radical hydrocarbon intermediate (or less likely carbocation for one of the KSH mechanistic variants) and M–OH species. In the next step, the radical hydrocarbon intermediate recombines with the OH radical forming the alcohol product. This also results in a two electron reduction of the metal complexes from [(Fe^IV^=O)heme]^+•^ to [(Fe^III^)heme] for CYP, from Fe^V^=O(OH) to Fe^III^OH or Fe^III^(OH)_2_ for KSH and from Mo^VI^=O to Mo^IV^ for S25DH. The occurrence of the radical hydrocarbon intermediate in case of molybdenum hydroxylase may be a substrate dependent phenomenon as QM:MM calculations for ethylbenzene dehydrogenase suggest formation of a carbocation intermediate (Szaleniec et al. 2014).

**Fig. 5:**
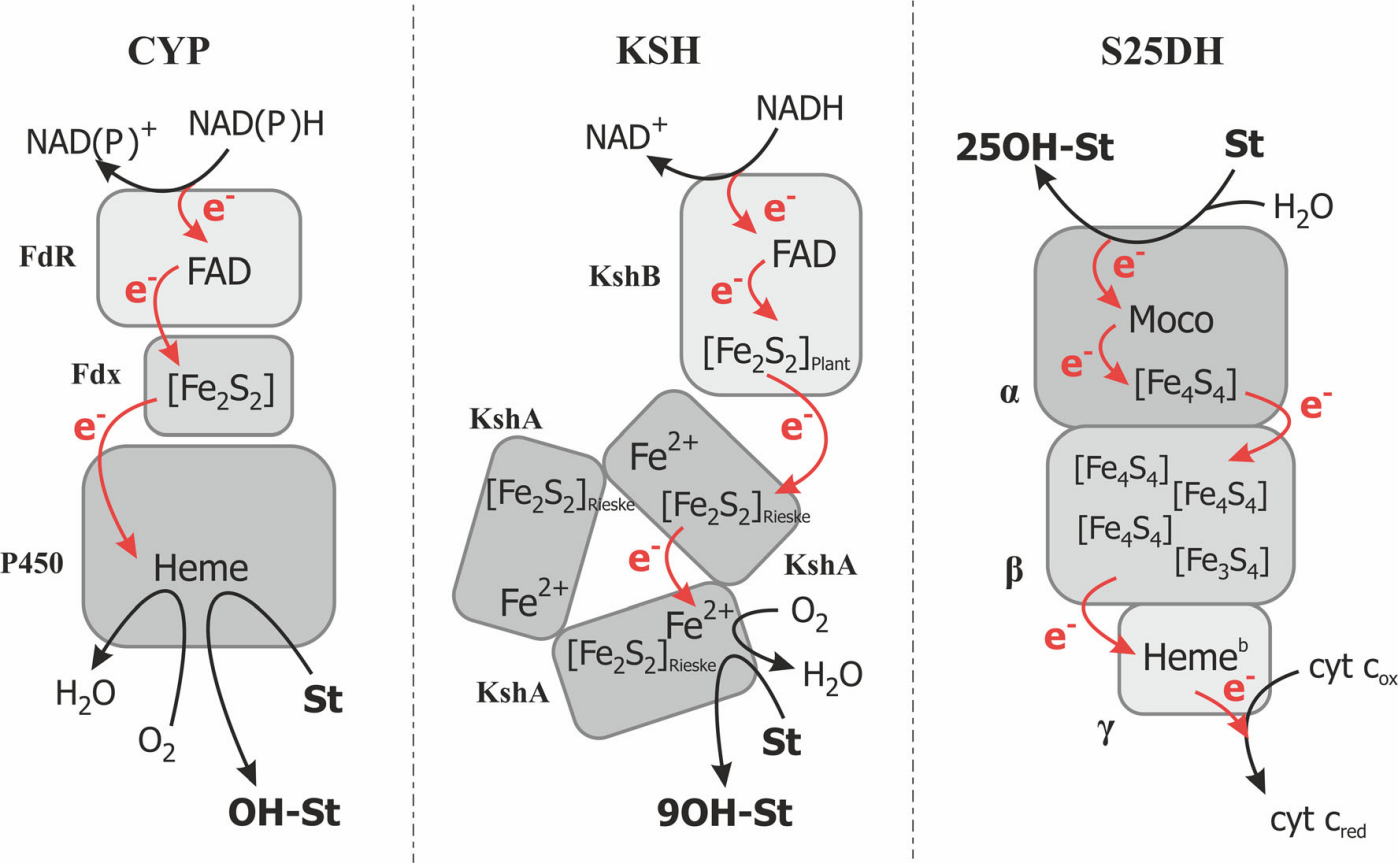
Schematic representation of electron transfer in various catalytic system hydroxylating steroids. CYP = scheme according to (Hannemann et al. 2007), FdR = ferrodoxin reductase, Fdx = ferrodoxin, P450 = cytochrome P450 monooxygenase; KSH according to (Petrusma et al. 2014), KshB = ferrodoxin reductase component, KshA = terminal oxygenase component; S25DH according to (Heider et al. 2016), α = catalytic subunit, β = electron transfer subunit, γ = subunit with heme b transferring electrons to external acceptor. In dark gray = catalytic subunits. St = steroid. Red arrow marks the electron transfer pathways

One of the main differentiating factors between CYP/KSH and S25DH is the nature of the available enzyme reoxidants. In the former case, the enzymes utilize molecular oxygen which, at the end, requires four-electron reduction (two electrons from the substrate and two electrons from the external source), whereas in the case of S25DH, two one-electron acceptors (most probably from bacterial cyt *c*) are employed for enzyme reoxidation (Fig. 5). In case of the O_2_-dependent hydroxylation, the additional two electrons have to be introduced by the external electron donors, such as NAD(P)H (via for example a ferredoxin and a ferredoxin reductase) in case of CYP or from NADH via the KshB subunit of Ksh. As a result, the O_2_ molecule is converted into H_2_O and an O^2−^ ligand, which is bound to the Fe atom.

In case of S25DH, the reoxidation of the catalytically inactive Mo^IV^–OH_2_ is carried out by two one-electron shifts via a chain of Fe–S cofactors coupled to heme in the γ subunit, which channels out electrons to the external electron acceptor, most probably cytochrome *c*. As a result, the CYP/KSH require electron inflow for the reaction to proceed, while in case of the molybdenum hydroxylase we observe only the electron outflow. This difference, however, stems solely from the chemical nature of the molecular oxygen, which without partial reduction, is quite unreactive. To some extent, one can treat molybdenum hydroxylases as an anaerobic equivalent of monooxygenases although the Mo^VI^=O seems to exhibit lower oxidation strength than that found in compound I or non-heme Fe-only complexes.

## Conclusion

Herein, we have described three biotechnologically important classes of bacterial steroid hydroxylases, namely cytochrome P450, Rieske-type KSH, as well as molybdenum steroid hydroxylase. For the first time in the literature, we have combined the available mechanistic studies for the Rieske-type oxygenases with structural data collected for KSH, which enabled formulation of the mechanistic hypothesis. Based on this, further studies, aimed at testing potential variants of the mechanism, can be more easily planned and executed.

We also discussed and compared available mechanistic data on all three classes of enzymes, which enabled us to delineate important similarities and unavoidable differences in their reaction mechanism. Despite the fact of a very different composition of the metal sites in the active centers of the discussed enzymes (heme, mono-Fe complex, Mo-cofactor), the reductive half-cycle responsible for steroid hydroxylation is almost identical for all three classes of enzymes and involves radical steroid intermediate. The existing differences are mainly associated with the oxidative half-cycle and originate from differences in the type of electron acceptor, i.e., O_2_ in the aerobic metabolism and heme *c* in anaerobic metabolism. In the former case, restoration of the catalytically active, high-valent Me–O species, requires additional two electrons that have to be delivered to the system in order to activate the otherwise inert oxygen molecule. This result with seemingly different electron flow through the enzymatic systems of P450 and Rieske-type monooxygenases than that observed for EBDH-like enzymes. However, beside that fact, there is always two electron flows from the steroid substrate to the metal cofactor.

Finally, we have summarized potential biotechnological applications of all three systems in the synthesis of valuable hydroxy steroid compounds. We believe that a critical mass of the knowledge was already reached and with recent development of the recombinant and/or the whole-cell biocatalytic systems we will soon see much wider implementation of the described enzymes in fine chemical and pharmaceutical industry.
